# RadialPheno: A tool for near‐surface phenology analysis through radial layouts

**DOI:** 10.1002/aps3.1253

**Published:** 2019-06-05

**Authors:** Greice C. Mariano, Bruna Alberton, Leonor Patrícia C. Morellato, Ricardo da S. Torres

**Affiliations:** ^1^ Institute of Computing University of Campinas Campinas, São Paulo Brazil; ^2^ Institute of Biosciences, Botany Department, Phenology Lab Universidade Estadual Paulista (UNESP) Rio Claro, São Paulo Brazil

**Keywords:** cyclical temporal data, information visualization, leafing, phenocameras, radial layouts

## Abstract

**Premise:**

Increasingly, researchers studying plant phenology are exploring novel technologies to remotely observe plant changes over time. The increasing use of phenocams to monitor leaf phenology, based on the analysis of indices extracted from sequences of daily digital vegetation images, has demanded the development of appropriate tools for data visualization and analysis. Here, we describe RadialPheno, a tool that uses radial layouts to represent time series from digital repeat photographs, and applies them to the analysis of leafing patterns and leaf exchange strategies of different vegetations.

**Methods and Results:**

We developed a web tool, RadialPheno, provided with the R and Shiny environments, which uses radial visual structures to represent cyclical multidimensional temporal data associated with digital image time series. We demonstrate the application of our methods and tool for a savanna vegetation phenology in the Brazilian Cerrado. We visually represented the greenness index extracted from sequential imagery using the RadialPheno tool.

**Conclusions:**

RadialPheno was successfully applied for the visualization and interpretation of individual, species, and community long‐term leafing phenology data associated with near‐surface phenological observations of Cerrado vegetation. RadialPheno was also effective for intercomparisons of ground‐based direct visual observations and camera‐derived phenology observations.

Plant phenology studies rely on the long‐term observation of individual plants and the associated environmental conditions (Schwartz, 2003; Morellato et al., 2016). Typical methodologies rely on the direct observation of plants on the ground (Morellato et al., 2010a). This approach, however, is a time‐consuming and error‐prone task, mainly due to the need of observing hundreds of individual plants for long periods of time (Morellato et al., 2016; Alberton et al., 2017). A suitable alternative relies on the use of remote monitoring techniques based on the use of sequential images taken by near‐surface digital cameras (Richardson et al., 2007, 2009; Alberton et al., 2014, 2017).

Near‐surface remote phenology uses sensors (i.e., digital cameras, also called phenocams) installed close to ground level (to monitor individual species leafing patterns) or at the top of towers (to monitor ecosystem‐scale vegetation leafing patterns). Examples of leafing patterns include leaf flushing and senescence, which may provide evidence of leaf exchange strategies for different species (e.g., deciduous, semi‐deciduous, evergreen) (Camargo et al., 2018). In the tropics, the high diversity of species and climatic conditions is reflected in a wide variety of leafing strategies present in the communities, with subjective interpretation based on visual direct observations (Alberton et al., 2017; Camargo et al., 2018). One of the main advantages of using sequential images to monitor plant phenology, in comparison with traditional ground‐based direct visual phenology, is that it provides high temporal resolution (allowing hourly or daily data collection) to detect leaf change strategies simultaneously across multiple sites, with reduced human effort for data acquisition and low cost (Morisette et al., 2009; Richardson et al., 2013, 2018; Alberton et al., 2017). However, at the same time, the high‐frequency data collected at a single or multiple sites have become increasingly complex, demanding the development of robust tools to assist the management and analysis of a large volume of multidimensional and multivariate time series data (e.g., Filippa et al., 2016; Leite et al., 2016; Mariano et al., 2017).

In this context, we propose a new tool to support the identification of recurrent life cycle events at individual, species, and community levels using a phenocam imagery time series. We chose the radial representation, which takes into account the recurrence of life cycle events and the absence of a true starting date, and is especially appropriate for tropical vegetation phenology lacking a dormant season (Morellato et al., 2010b; Mariano et al., 2017; Camargo et al., 2018). Aigner et al. (2011) introduced several information visualization approaches concerning the presentation of time‐related data. Few of those approaches are appropriate for phenology studies, as they often do not handle multidimensional temporal data, which are both long and cyclical. RadialPheno offers a different approach. It is a temporal visualization system based on radial layouts that supports visual identification of temporal changing patterns associated with a phenological multidimensional cyclical time series. We adopted the idea of concentric circles presented by Daassi et al. (2000) to encode the time dimension and show the multivariate temporal data within concentric circles, combining the circle view presented by Keim et al. (2004) with circle segments presented by Ankerst et al. (1996). This approach allows for the direct comparison of values associated with multiple variables along cyclical temporal seasons. In addition, the radial visualization is more appropriate for tropical vegetation where the growing season is continuous with no marked resting season. Radial visualization better illustrates the continuous leafing patterns, leaf exchange strategies, and peaks of activity (Morellato et al., 2010b).

In a previous study, we demonstrated through a set of user tests that radial layouts show potential to encode cyclical phenological data associated with ground‐based visual observations (Mariano et al., 2018). Our evaluation also suggests that multiple interaction possibilities (e.g., filtering and selection options) should be implemented to support the customization of the data presented.

We demonstrated the application of our proposed visualization approach for encoding data extracted from digital images taken from the Cerrado, a Brazilian Neotropical savanna, one of the monitoring sites of the e‐phenology network (www.recod.ic.unicamp.br/ephenology) (Alberton et al., 2014, 2017; Mariano et al., 2016). To the best of our knowledge, this is the first work in the literature describing the use of radial visualization to illustrate phenocam data, using daily temporal series green chromatic coordinate (Gcc) data extracted from repeated digital images..

## METHODS AND RESULTS

RadialPheno was designed as a web application using R and Shiny (www.shiny.rstudio.com). R is a free software environment for statistical computing, while Shiny is an R package used to build interactive web interfaces and dashboards. The interface was developed and tested on a Windows operating system and is distributed under a GNU license. RadialPheno is available on GitHub (https://github.com/gcmariano/radialPheno), with links to the source code, a time series example, and documentation.

**Figure 1:**
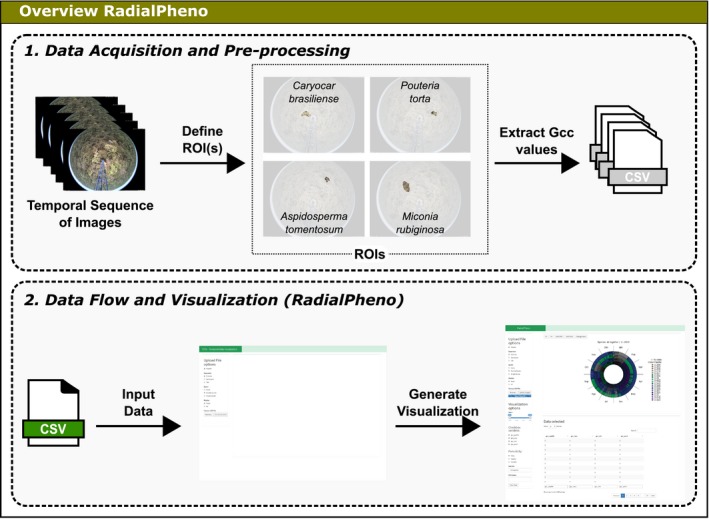
Example of RadialPheno workflow starting with a set of images from a fisheye phenocam and four regions of interest (ROIs); each ROI represents one individual species' crown. From the image processing, indices including the daily green chromatic coordinate (Gcc) values representing the leaf flushing phenophases for each species are obtained and saved as a CSV file, which is used as a data input in RadialPheno. The CSV files must be composed of year, month, doy, and at least one variable varying over time.

We illustrate the use of RadialPheno to visualize the greenness index extracted from sequential imagery of Cerrado vegetation located in southeastern Brazil (Alberton et al., 2014). The process of digital image analysis depends on the definition of regions of interest (ROI), representing a region within the sample image defined for analysis (see Richardson et al., 2007; Alberton et al., 2014, 2017). An ROI represents an individual tree crown (our study case), populations, or a community or vegetation type within the landscape (Richardson et al., 2009; Alberton et al., 2017). From each ROI, we extract vegetation indices from red, green, and blue (RGB) color channels of the digital images (Richardson et al., 2007; Sonnentag et al., 2012; Filippa et al., 2016). For instance, the Gcc index is the normalized green amount present in a digital image (for RGB chromatic coordinates, see Woebbecke et al., 1995). The Gcc index represents a measure of vegetation greenness and has been widely used to characterize seasonal changes in leaf phenological stages, such as new leaves, leaf expansion, and senescence (Richardson et al., 2007; Sonnentag et al., 2012; Alberton et al., 2014), as well as physiological properties, such as leaf area index (LAI), chlorophyll content, and gross primary productivity (Ahrends et al., 2009; Migliavacca et al., 2011; Toomey et al., 2015). In this work, we selected ROIs corresponding to the individual crown of four Cerrado species—*Aspidosperma tomentosum* Mart., *Caryocar brasiliense* Cambess., *Miconia rubiginosa* (Bonpl.) DC., and *Pouteria torta* (Mart.) Radlk. (Fig. 1)—from which we extracted daily Gcc values (Alberton et al., 2014; Almeida et al., 2016; Filippa et al., 2016). After the image processing stage, the information about the time and the greenness index for each species, hereafter referred to as *gcc_aspido*,* gcc_cary*,* gcc_mic*,* gcc_pout*, was saved as a set of CSV files (Fig. 1).

CSV files must follow a specific format: a set of three variables to identify the time scale (where columns must be named as *year*,* month*, and *doy*) and one or more variables that correspond to the phenological information. In our example, we are using the greenness index for four different species (as explained above). RadialPheno supports the visualization of time series organized in single or multiple CSV files (Fig. 1). The next step is the visualization of the processed data (CSV files).

**Figure 2:**
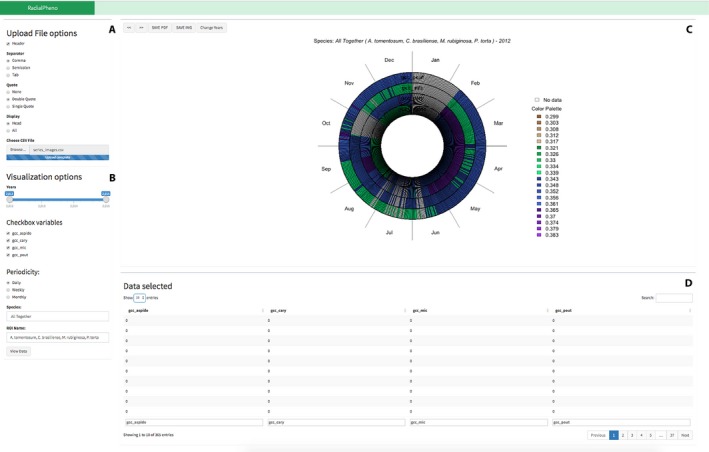
Screenshot of RadialPheno filters (A, B) and visualization (C, D) after the user uploads a file with the columns: year, doy, gcc_aspido, gcc_cary, gcc_mic, gcc_pout. Data refers to the green chromatic coordinate (Gcc) computed from daily images taken by digital cameras for the species *Aspidosperma tomentosum*,* Caryocar brasiliense*,* Miconia rubiginosa*, and *Pouteria torta*, during the years 2012–2015.

In the initial interface of RadialPheno, users access the main filters to define the period and variables of interest. Then, users are expected to upload the data (CSV file); according to the attributes presented in the file, selectable filters will appear (Fig. 2A). In our example, when uploading the file with Gcc series for the four different species, the interface shows the Gcc attributes as selectable filters, and the user can select the desired variables to be visualized (Fig. 2B). After selecting the options, the next step is to click the *View Data* button, which processes the input data and draws a radial structure that combines all of the selected variables (Fig. 2C). Once the visualization is presented, the user can navigate in the visualization over years, using the buttons *previous* (<<) and *next* (*>>*), and save the image as a document (PDF) or image (PNG) file, using the *Species* and *ROI Name* fields to name the files and the visualization. When only one variable has been selected, the user also has the option to click the *Change Years* button to visualize all years together (i.e., all years in one radial structure), avoiding movement between years by using the navigation buttons (see also Appendix S1: Figs. S1, S2, and S3). The ROI selection contributes to the analysis of the species leafing phenological patterns. In addition, the system identifies the time‐related columns in the file and presents the annual and daily data at monthly timescales (Fig. 2A). In terms of the data organization, years are represented by circles and segments within the circle represent months, totaling 12 segments (months) that can be subdivided according to the timescale considered (e.g., daily, weekly) (Fig. 1). For example, for our daily image data, it is possible to select the timescale to visualize the data at weekly or monthly intervals (see Appendix S1: Fig. S4). The distribution can be changed according to the users' goals, so that circles can represent years or other variables (e.g., Gcc, red chromatic coordinate [Rcc], ROI) (see Appendix S1: Fig. S5 for the representation of two ROIs from the same species).

Finally, the interface can manage the phenological visualization of direct, on‐the‐ground observations, enabling the user to compare and analyze ground‐based visual phenological records and camera‐derived data all together (see Appendix S1: Fig. S6). In this case, the CSV file should contain both direct observation data and phenocam data. Another option is to analyze the ground‐based phenological observations separately. In this case, the CSV file would contain only data related to the direct observations.

## CONCLUSIONS

We developed an innovative web application using the R language and Shiny framework to support phenology researchers with daily data analysis, offering appropriate visualization approaches and facilitating the investigation of the evolution of cyclical phenomena over time and the visualization of complex temporal change patterns or even correlations among multidimensional variables. The introduced visualization tool, RadialPheno, uses radial visual structures to present cyclical multidimensional temporal data associated with phenocam near‐surface remote phenology.

We tested RadialPheno using real data collected from the Brazilian Cerrado, a seasonal Neotropical savanna habitat, and vegetation indices obtained by daily phenocam images. Finally, we discuss the use of RadialPheno for ground‐based direct visual phenology observation data of the same study site, demonstrating its potential for comparison of these two monitoring approaches and, consequently, its usefulness for validation of phenocam‐derived leaf phenology.

We have identified a few limitations for RadialPheno: (1) the visualization of phenological data using multiple segments, which may hinder the proper identification of cyclical temporal data patterns in inner circles due to size constraints, and (2) the need for additional interaction/control mechanisms to support the selection of data of interest using database‐oriented technologies (Mariano et al., 2016) and the selection of visualization settings (e.g., color palette definition, normalization functions).

We will continue further developments and improvements of RadialPheno. One planned improvement is the integration of circular statistical analysis (e.g., calculations of mean date and concentration of phenophases to access the degree of seasonality) and phenological metrics (e.g., start and peak dates of leaf phenophases) (Morellato et al., 2000, 2010b; Camargo et al., 2018).

## Supporting information


**APPENDIX S1.** A RadialPheno case study using phenological on‐the‐ground direct observations and phenocam data.

## Data Availability

RadialPheno is available on the GitHub repository (https://github.com/gcmariano/radialPheno), with links to the source code, a time series example, and documentation.
