# Supplementary material for: RadialPheno: A tool for near‐surface phenology analysis through radial layouts
Source: Appl Plant Sci. 2019 Jun 5;7(6):e01253. doi: 10.1002/aps3.1253 (PMC6580983; doi:10.1002/aps3.1253)
Supplement: Supplementary file 1 — APPENDIX S1. A RadialPheno case study using phenological on‐the‐ground direct observations and phenocam data. [file APS3-7-e01253-s001.pdf]

DOI

APPENDIX S1. A RadialPheno case study using phenological on-the-ground direct observations and phenocam data.

In our paper, we describe the functional overview of RadialPheno in the context of digital images taken for remote monitoring of phenology. Here, we provide an example of the use of RadialPheno using a CSV file with the following columns: *year*, *doy*, *gcc_aspido*, *gcc_cary*, *gcc_mic*, *gcc_pout*, with green chromatic coordinate (Gcc) data computed for the species *Aspidosperma tomentosum*, *Caryocar brasiliense*, *Miconia rubiginosa*, and *Pouteria torta* during the years 2012 to 2015. This case study explores the use of the tool in greater depth and demonstrates how the data can be exploited from different perspectives. We also include a new real-world usage scenario for RadialPheno: the visualization of on-the-ground direct observations. We also provide a visual comparison between the direct observations and the image data.

Suppose that a user wants to analyze how the Gcc vegetation index obtained from sequences of images using masks for the species *A. tomentosum* evolves over time, during the period from 2012 to 2015 (Fig. S1). The user needs to select the desired variable and click the *View Data* button. As explained in our paper, the user can visualize the data for different years using the navigation buttons (previous and next). Another option is to click the *Change Years* button to visualize all years together (i.e., all years in one visualization; Fig. S2).

The same selection and options can be done for the other variables separately. For example, Figure S3A–C presents the daily Gcc values for the species *C. brasiliense*, *M. rubiginosa*, and *P. torta*, respectively. In this case, we can compare the different species. Note, for example, that *M. rubiginosa* and *P. torta* have similar scores over time, and the range of those scores is greater than that of *C. brasiliense*, which presents intensities concentrated near the highest scores (around 0.33).

Going back to the initial interface, the user may select all Gcc values to be visualized for one year using the weekly or monthly filters (in addition to the daily option). See Figure S4A–C for examples of visualizations generated using these filters for daily, weekly, and monthly summarization, respectively.

In summary, as we can observe, the data organization within RadialPheno concerning time information works as follows: circles are associated with years, while segments within the circle are used to represent months. Therefore, there are 12 segments that can be subdivided according to what timescale is considered (e.g., daily, weekly, or monthly). However, it is important to note that this distribution can be changed according to the user's goals, so that the circles can represent years or other variables (e.g., Gcc, means of RGB channels, regions of interest [ROIs]).

This kind of visualization and the development of RadialPheno was guided mainly by three target phenological research questions, as follows:

1. How to visually detect seasonal patterns at both the individual and species levels? How can vegetation indices be used to support such analysis?
2. How to visually compare data acquired from individuals of the same species by near-remote phenology? How to visually compare life cycles of individuals from different species (at species level)?
3. How to visually correlate on-the-ground phenology data with near-remote phenology? How to deal with the different timescales?

For the first question, which was related to the visualization of seasonal patterns at the individual and species levels, we explored the use of the Gcc vegetation index as the main attribute to visualize the temporal properties from images so that data about individuals and species can be compared (e.g., as illustrated in Figs. S1 and S2). However, the tool is flexible enough to take other measures and attributes, such as those shown in Figure S5. In this case, we show data from two different individuals (e.g., ROIs) for the species *Myrcia guianensis* during 2015. In this example, this kind of analysis is related to the second research question.

Finally, we investigated the potential use of RadialPheno to visualize on-the-ground, direct observation phenological data (as illustrated in Fig. S6), and the potential to compare with camera-derived, near-remote phenology data. In Figure S6, we plot both on-the-ground phenological data (represented as the percentage of individuals leafing out) and near-remote Gcc vegetation index for the species *A. tomentosum*, *C. brasiliense*, *M. rubiginosa*, and *P. tomentosa*, by month during the year 2015 (addressing the third research question). In this case, we separate the ground and camera data as two visualizations, but it is also possible to integrate the data into a single visualization, using a normalization procedure to set a suitable range of values for both kinds of data.

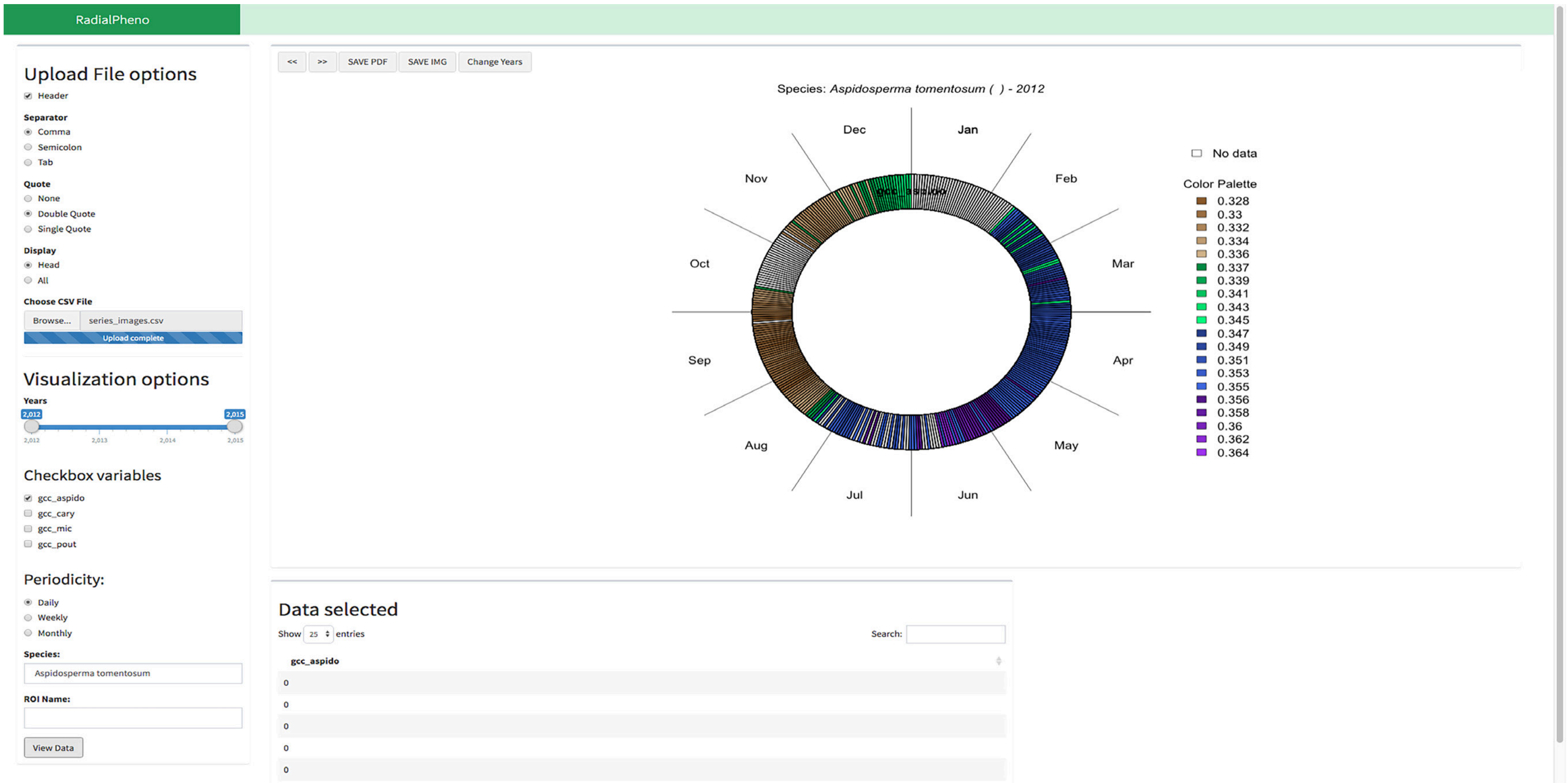

FIGURE S1. Screenshot after the user selects the *gcc_aspido* variable to visualize. The visualization shows the Gcc values for *Aspidosperma tomentosum* for the year 2012. The user can navigate through the years using the navigation buttons on the top left of the tool interface.

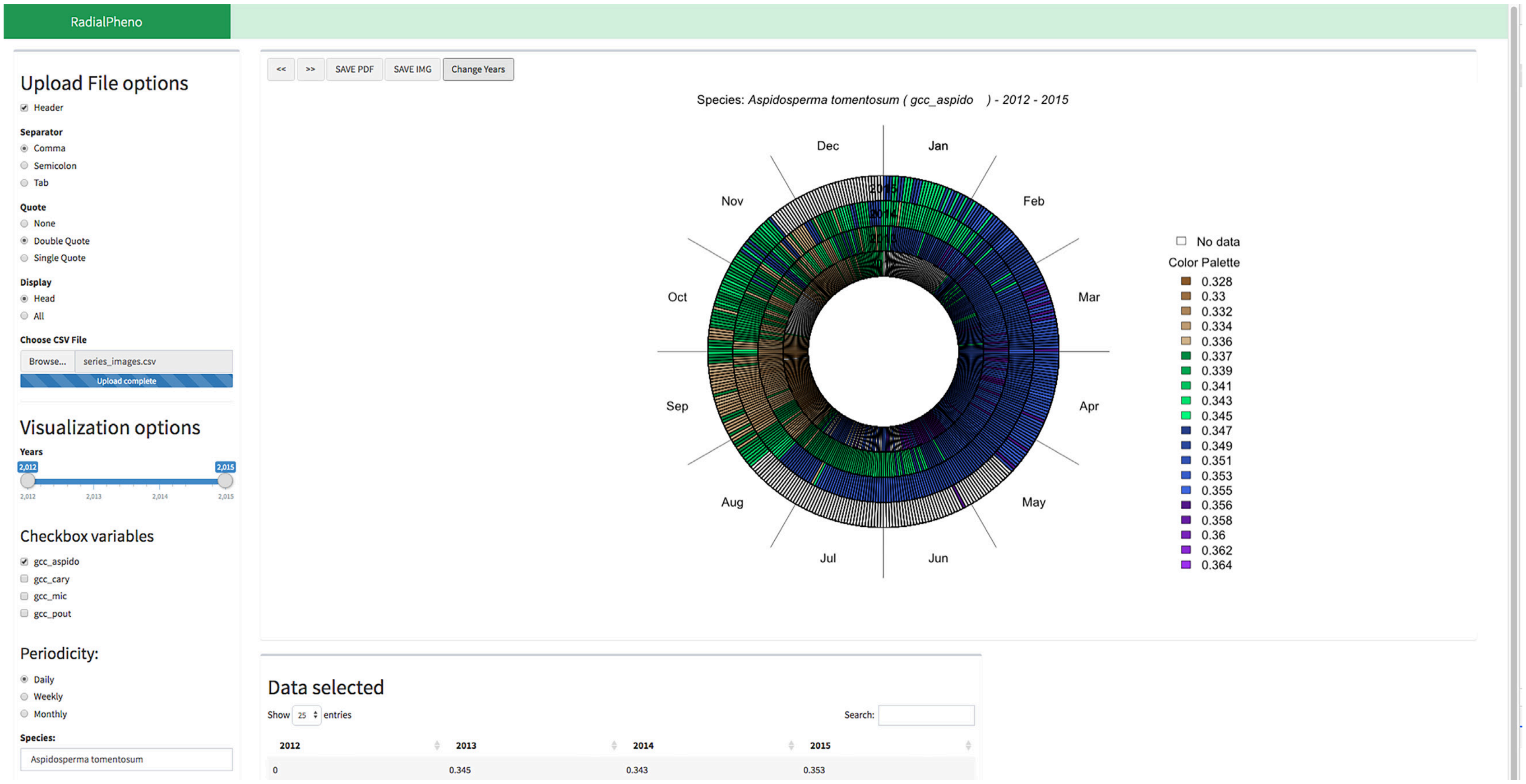

FIGURE S2. Screenshot after the user clicks on the *Change Years* button (located on the top of the visualization). The visualization changes to represent the daily Gcc values for *Aspidosperma tomentosum* for all years combined. Each year is represented by an inner circle.

DOI

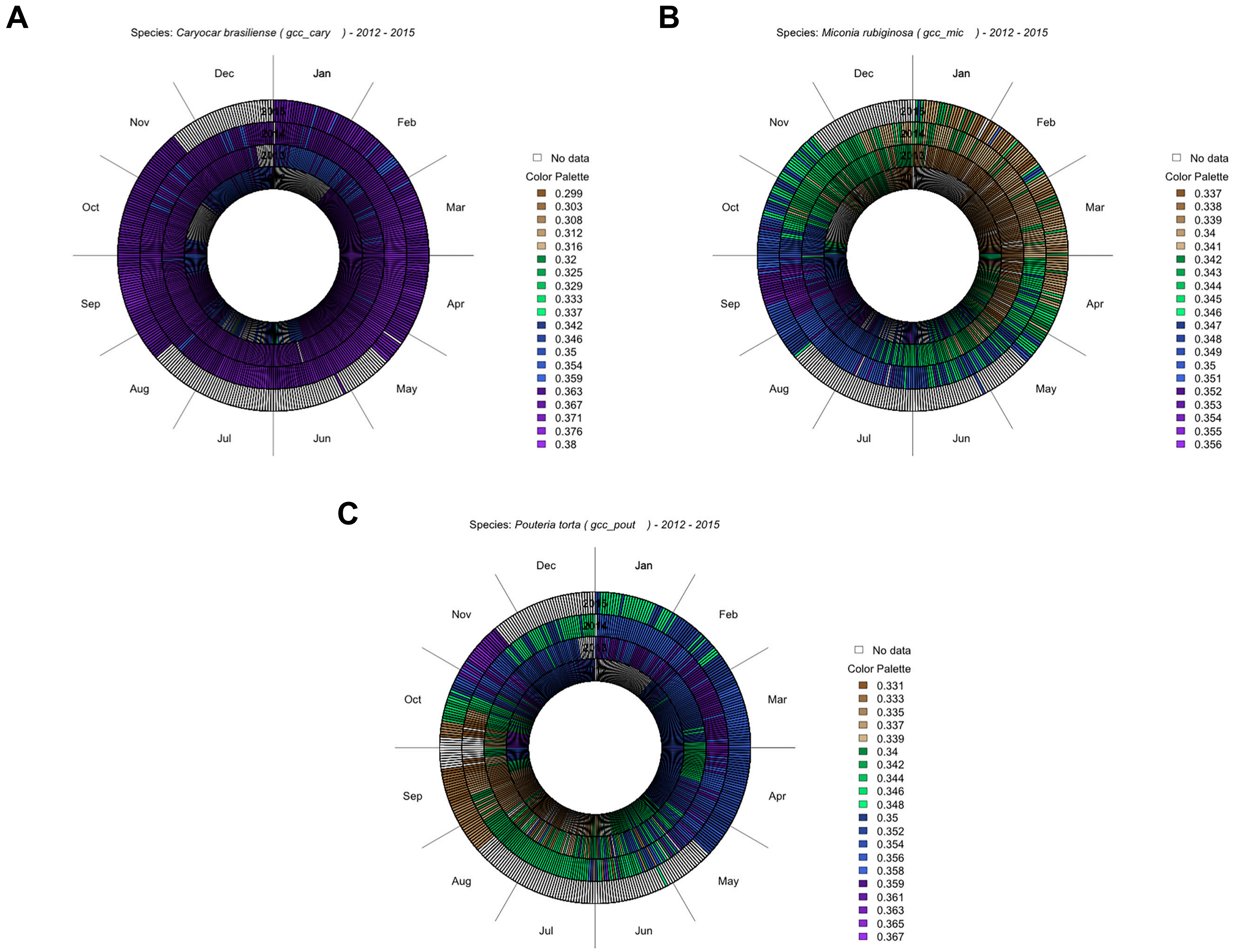

FIGURE S3. Radial structures encoding the daily Gcc values over individual months of the years 2012 to 2015 (inner circles), extracted for one individual crown of three species: (A) *Caryocar brasiliense*, (B) *Miconia rubiginosa*, and (C) *Pouteria torta*.

DOI

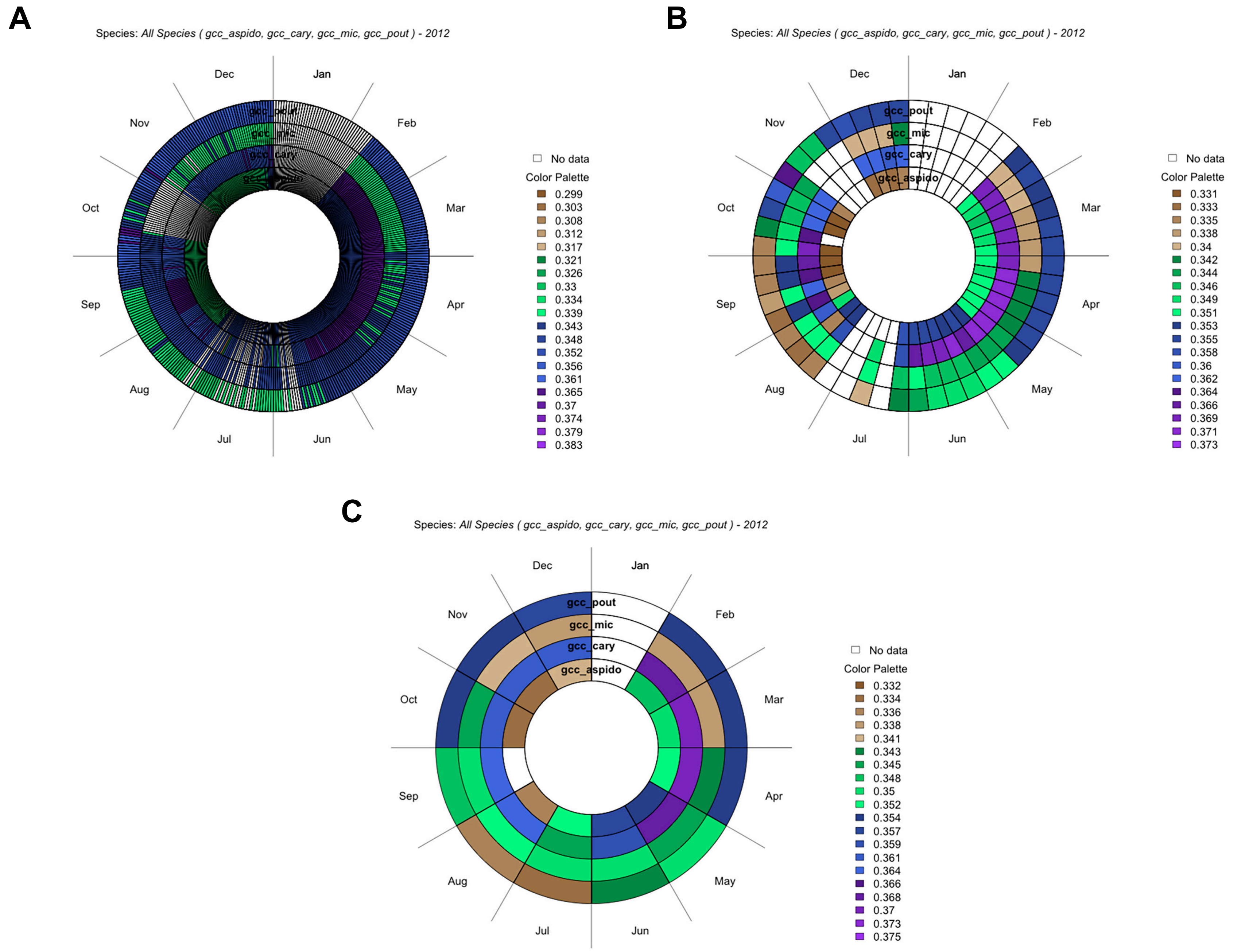

FIGURE S4. Radial structures encoding the (A) daily, (B) weekly, and (C) monthly Gcc values during the year 2012, observed for individuals of four species, from the inner circle toward the outer circle: *Aspidosperma tomentosum*, *Caryocar brasiliense*, *Miconia rubiginosa*, and *Pouteria torta*.

DOI

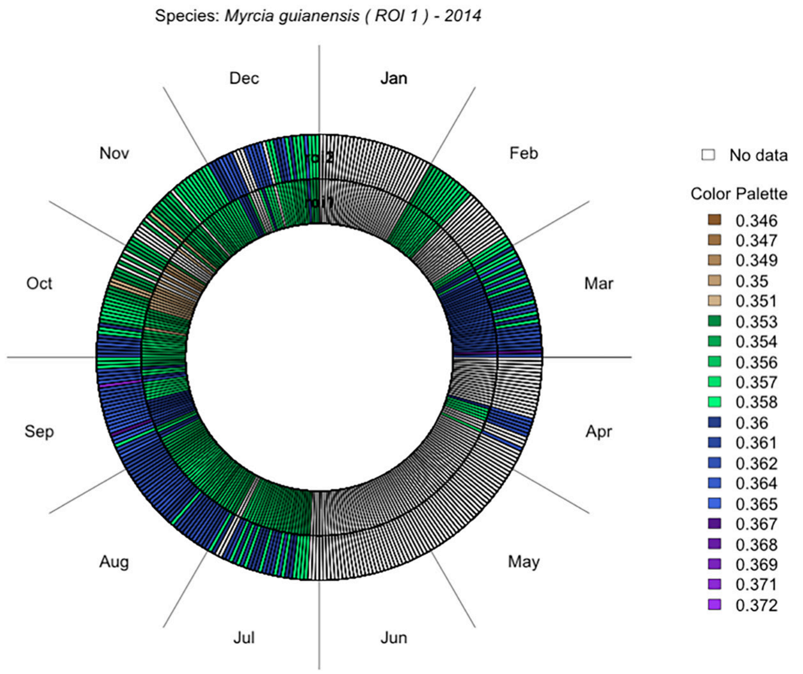

FIGURE S5. Radial structure encoding the Gcc values for two regions of interest (individual crowns) of the species *Myrcia guianensis*.

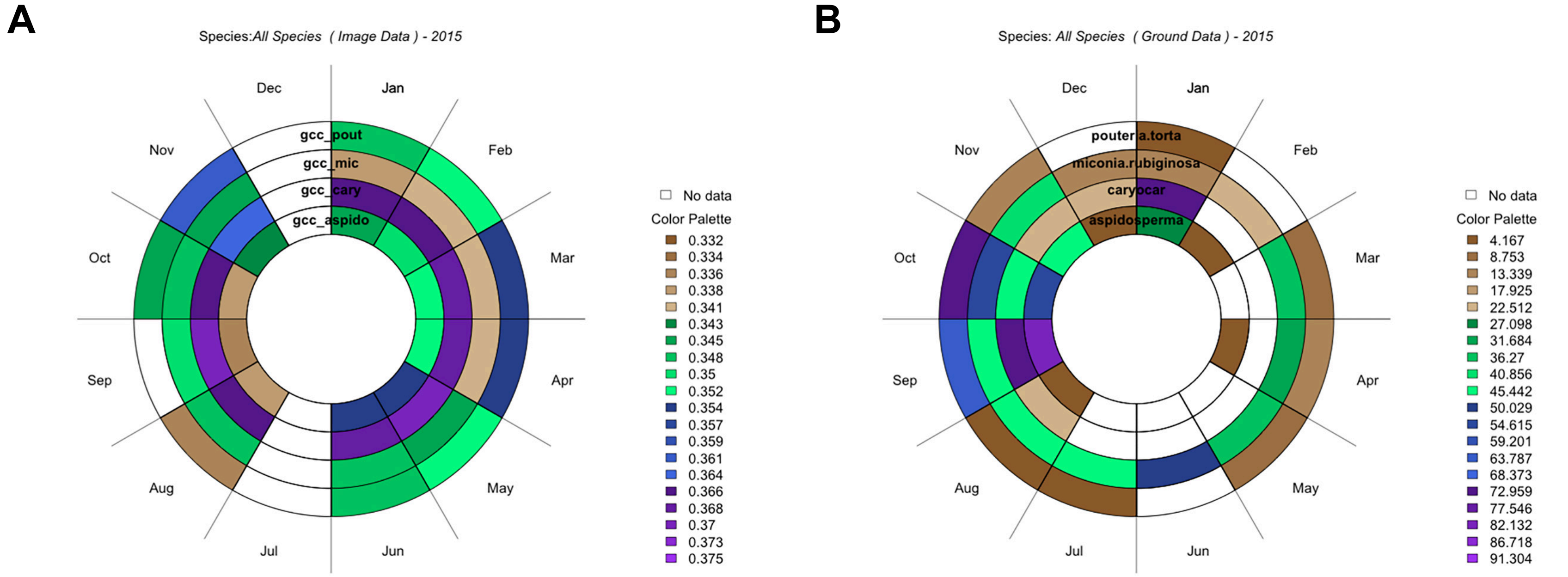

FIGURE S6. Radial structures encoding the camera-derived Gcc index (A) and direct on-the-ground leaf out (B) phenology observations for the species *Aspidosperma tomentosum*, *Caryocar brasiliense*, *Miconia rubiginosa*, and *Pouteria tomentosa* by month, during the year 2015.
